# Mediation of the APOE Associations With Cognition Through Cerebral Blood Flow: The CIBL Study

**DOI:** 10.3389/fnagi.2022.928925

**Published:** 2022-06-30

**Authors:** Yan-Li Wang, Mengfan Sun, Fang-Ze Wang, Xiaohong Wang, Ziyan Jia, Yuan Zhang, Runzhi Li, Jiwei Jiang, Linlin Wang, Wenyi Li, Yongan Sun, Jinglong Chen, Cuicui Zhang, Baolin Shi, Jianjian Liu, Xiangrong Liu, Jun Xu

**Affiliations:** ^1^Department of Neurology, Beijing Tiantan Hospital, Capital Medical University, Beijing, China; ^2^China National Clinical Research Center for Neurological Diseases, Beijing Tiantan Hospital, Capital Medical University, Beijing, China; ^3^Department of Cardiology, Weifang People’s Hospital, Weifang Medical University, Weifang, China; ^4^Institute of Translational Medicine, Medical College, Yangzhou University, Yangzhou, China; ^5^Department of Neurology, Peking University First Hospital, Peking University, Beijing, China; ^6^Division of Neurology, Department of Geriatrics, National Clinical Key Specialty, Guangzhou First People’s Hospital, School of Medicine, South China University of Technology, Guangzhou, China; ^7^Department of Neurology, The Second Affiliated Hospital of Xuzhou Medical University, Xuzhou, China; ^8^Department of Neurology, Weifang People’s Hospital, Weifang Medical University, Weifang, China; ^9^Department of Neurology, Fuxing Hospital, Capital Medical University, Beijing, China

**Keywords:** APOE ε4, cerebral blood flow, cognition, causal mediation, CIBL study

## Abstract

**Background:**

The ε4 allele of the apolipoprotein E (APOE) gene is a strong genetic risk factor for aging-related cognitive decline. However, the causal connection between ε4 alleles and cognition is not well understood. The objective of this study was to identify the roles of cerebral blood flow (CBF) in cognitive-related brain areas in mediating the associations of APOE with cognition.

**Methods:**

The multiple linear regression analyses were conducted on 369 subjects (mean age of 68.8 years; 62.9% of women; 29.3% of APOE ε4 allele carriers). Causal mediation analyses with 5,000 bootstrapped iterations were conducted to explore the mediation effects.

**Result:**

APOE ε4 allele was negatively associated with cognition (*P* < 0.05) and CBF in the amygdala, hippocampus, middle temporal gyrus, posterior cingulate, and precuneus (all *P* < 0.05). The effect of the APOE genotype on cognition was partly mediated by the above CBF (all *P* < 0.05).

**Conclusion:**

CBF partially mediates the potential links between APOE genotype and cognition. Overall, the APOE ε4 allele may lead to a dysregulation of the vascular structure and function with reduced cerebral perfusion, which in turn leads to cognitive impairment.

## Introduction

According to World Alzheimer Report 2019, the number of people with dementia is over 50 million currently, which is projected to be 82 million in 2030 and 152 million in 2050 (Sheet, 2019). According to the latest epidemiological survey, the incidence of mild cognitive impairment is 15.5% among people aged over 60 years in China (Jia et al., 2020). The vascular factor and apolipoprotein E gene (APOE) ε4 allele are important factors associated with cognitive impairment (Bretsky et al., 2003; Strickland, 2018). Previous studies have drawn conflicting conclusions concerning the association between CBF and cognition. Some argued that reduced cerebral blood flow (CBF) is independently associated with worse cognitive performance (Bracko et al., 2020; Visser et al., 2020), especially, in the hippocampus, posterior cingulate, precuneus, thalamus, and caudate (Nation et al., 2013; Okonkwo et al., 2014). However, Steffener et al. suggested that cognition is negatively correlated with CBF in the posterior central gyrus, hippocampus, and part of the temporal cortex. Therefore, the relationship between cognition and CBF still needs further exploration. Besides, the relationship between CBF and APOE is still contradictory (Zlatar et al., 2014; Michels et al., 2016; Dounavi et al., 2021). The PREVENT-Dementia study shows that CBF is higher in ε4 carriers than in non-carriers across the general population (Dounavi et al., 2021), whereby a small-sample study of 48 subjects finds an inverse relationship between CBF and APOE ε4 (Michels et al., 2016). Moreover, the relationship is inconclusive at different stages of Alzheimer’s disease (AD) (Wierenga et al., 2012; Kim et al., 2013; Michels et al., 2016; McKiernan et al., 2020). CBF is significantly increased in the cognitively normal (CN) ε4 carriers, (Wierenga et al., 2012; McKiernan et al., 2020) and decreased in mild cognitive impairment (MCI) ε4 carriers (Wierenga et al., 2012). However, the results are exactly the opposite in an age-matched cohort study (Kim et al., 2013). However, it has been widely accepted that APOE ε4 is the most common genetic risk factor for cognitive decline, and the mechanism underlying ε4 allele effects on cognition is not clear. Previous mediation analyses reveal that APOE ε2 may exert a protective effect on neurofibrillary tangles by two pathways: a direct effect of the ε2 allele (direct pathway) and *via* its effect on neuritic plaques (indirect pathway) (Serrano-Pozo et al., 2015). Thus, it could be speculated that the ε4 allele exerts its effects on cognition by both direct and indirect pathways. To date, the roles of CBF on the associations of APOE genotype with cognition have not been studied carefully. Herein, we aimed (1) to explore the relationships of APOE genotype with CBF and cognition and (2) to test whether the influences of APOE genotype on cognition is mediated by CBF.

## Materials and Methods

### The Chinese Imaging, Biomarkers and Lifestyle Database

The Chinese Imaging, Biomarkers and Lifestyle (CIBL) Study of Alzheimer’s Disease is an ongoing large-scale study mainly focused on radiographic changes, biomarkers, and risk factors of AD, aiming to construct prediction models for early diagnosis of AD. The samples were recruited at Beijing Tiantan Hospital Affiliated with Capital Medical University since September 2020. All enrolled participants underwent neuroimaging examination, blood collection, and clinical and neuropsychological assessments *via* a structured questionnaire. The clinically cognitive diagnoses of MCI and AD were aligned with the National Institute on Aging-Alzheimer’s Association (NIA-AA) criteria (Albert et al., 2011; McKhann et al., 2011). Exclusion criteria for this study were (1) education illiterate groups; (2) cognitive impairment attributable to alcohol use, depression, medication use, or medical illness; (3) magnetic resonance imaging (MRI) contraindications; (4) using drugs or substance that affected cerebral perfusion on the same day as the MRI; (5) history of significant psychiatric disorder or neurological disease (e.g., central nervous system infection, traumatic brain injury, epilepsy, or other major neurological disorders); (6) life-threatening somatic disease; and (7) family history of Mendelian inheritance. All participants provided written informed consent prior to enrollment in the CIBL study, which was approved by the Research Ethics Committee of Beijing Tiantan Hospital in accordance with the Declaration of Helsinki.

### Participants

In this study, participants who had data seen as extreme values (situated outside ± 3 standard deviations) were removed from the analysis. Finally, 369 participants without a history of stroke or other structural brain abnormality were included in the CIBL study. Individual information including gender, age, APOE ε4 genotyping, educational level, systolic blood pressure (SBP), body mass index (BMI), history of hypertension, Mini-Mental State Examination (MMSE), Montreal Cognitive Assessment (MoCA), and CBF values was derived from arterial spin labeling (ASL). Age status was categorized as midlife (40 < age < 65) and late-life (age ≥ 65).

### Apolipoprotein E Genotypes and Cognitive Assessment

DNA samples were extracted from 10 ml ethylene diamine tetraacetic acid (EDTA) overnight fasting blood samples. All individuals were genotyped at WeGene Lab using a customized Illumina WeGene V3 Array by Illumina iScan System, which contains roughly 700,000 markers. APOE genotypes comprising ε2, ε3, and ε4 alleles were defined by single nucleotide polymorphisms (SNPs) rs429358 and rs7412. Participants in this study were classified as APOE ε4 non-carriers and APOE ε4 carriers (presence of at least one APOE ε4 allele). Global cognitive function was assessed for all participants using the MoCA test (Hemmy et al., 2020).

### Brain Magnetic Resonance Imaging

All participants in this study underwent brain MRI using a 3.0-T MR scanner (SIGNA Premier; GE Healthcare, Milwaukee, WI, United States) with the 48-channelhead coil. The imaging parameters for high-resolution three-dimensional (3D) T1-weighted scans were as follows: repetition time, 1,900 ms; echo time, 3.0 ms; flip angle, 12°; slice thickness, 1.0 mm; number of slices, 176; field of view, 256 × 256 mm^2^; acquisition matrix, 256 × 256; and scan time, 4 min 56 s. The perfusion-weighted MRI was performed using 3D pseudocontinuous arterial spin labeling (pCASL) sequences.

The acquisition parameters for eASL were as follows: repetition time, 4,849 ms; echo time, 10.6 ms; field of view, 220 × 220 mm^2^; acquisition matrix, 512 × 512; slice thickness, 4 mm; number of slices, 36; and scan time, 4 min 22 s. CBF of PLD (2,025 ms) was calculated according to the standard one-compartment model (Alsop et al., 2015). The perfusion regions of interest (ROIs) included the amygdala, hippocampus, parahippocampal gyrus, middle temporal gyrus, posterior cingulate, precuneus, and thalamus.

### Statistical Analyses

The statistical analyses and figure preparation were performed using SPSS version 24.0, R version 4.0.3, and GraphPad Prism version 8.0. According to the APOE genotypes, subjects were categorized into APOE ε4 carrier and non-carrier groups, and *t-*test (for continuous variables) and chi-square test (for categorical variables) were used to test the difference of baseline between-group characteristics. *P*-values were corrected for multiple hypotheses using the Benjamini–Hochberg method (Klipper-Aurbach et al., 1995).

First, multiple linear regressions (MLRs) were used to explore associations of cognition with APOE genotypes and CBF averaged across left and right hemispheres, adjusting for different covariates. Then, to assess the influence of hemispheric dominance on cognitive ability, CBF of both left and right hemispheres was included in the same MLR model (DELETED). Furthermore, to examine whether cerebral perfusion could modulate the relationship between APOE and cognition, causal mediation analyses were conducted based on the method suggested by Baron and Kenny (Baron and Kenny, 1986). The relative indirect effects (β_*IE*_) through CBF, natural direct effect (β_*DE*_), total effect (β_*total*_), and proportion of mediation (β_*IE*_/β_*total*_) were analyzed using bootstrapping with 5,000 iterations. A two-tailed *P*-value less than 0.05 was considered statistically significant.

## Results

### Description of the Subjects

Patient demographics and baseline characteristics are shown in Table 1. A total of 369 subjects were recruited for the study, including 92 CN, 124 MCI, and 153 Alzheimer’s dementia. The mean age was 68.82 ± 11.24 years and 62.87% were women. Of these, 108 (29.26%) were APOE ε4 allele carriers (≥ 1 ε4 allele). Compared with APOE ε4 non-carriers, APOE ε4 carriers were less educated (*P* = 0.02) (Araque Caballero et al., 2018) and performed significantly less well on the MMSE and the MoCA (*P* < 0.0001). No differences were registered in terms of age, gender, blood pressure, BMI, and history of hypertension (*P* > 0.05).

**TABLE 1:** Characteristics of subjects.

	All subjects	APOE ε4 non-carriers	APOE ε4 carriers	*P*-value	FDR_BH
	(*N* = 369)	(*N* = 261)	(*N* = 108)		
Female (N, %)	232,62.87	165,63.22	67,62.04	0.831^a^	
Age, years	68.82 ± 11.24	64.2 ± 11.53	66.31 ± 10.43	0.102	
Education, years	10.74 ± 4.56	11.11 ± 4.33	9.86 ± 5.00	**0.02**	
MMSE score	21.97 ± 7.58	23.22 ± 7.03	18.94 ± 8.03	**6.92E-07**	
MoCA score	17.19 ± 7.96	18.49 ± 7.51	13.95 ± 8.15	**6.03E-07**	
SBP, mmHg	128.62 ± 17.88	127.68 ± 17.07	131.38 ± 19.94	0.124	
BMI, kg/m^2^	23.92 ± 3.33	24.54 ± 9.48	23.83 ± 3.34	0.461	
Hypertension (N, %)	138, 37.40	100,38.61	38,36.19	0.666^a^	
**Regional CBF, mL/100 mg per minute**					
Amygdala_L	37.12 ± 7.89	37.61 ± 7.7	35.95 ± 8.27	0.067	0.094
Amygdala_R	35.76 ± 7.74	36.27 ± 7.74	34.52 ± 7.64	0.05	0.078
Hippocampus_L	39.92 ± 8.34	40.5 ± 8.07	38.54 ± 8.84	**0.041**	0.071
Hippocampus_R	39.27 ± 8.87	39.88 ± 8.46	37.79 ± 9.69	**0.04**	0.071
ParaHippocampal_L	36.54 ± 7.37	36.86 ± 7.24	35.76 ± 7.66	0.193	0.225
ParaHippocampal_R	38.2 ± 8.25	38.69 ± 8.04	37.02 ± 8.67	0.076	0.097
Temporal_Mid_L	48.8 ± 13.26	49.99 ± 12.96	45.91 ± 13.61	**0.007**	**0.028**
Temporal_Mid_R	44.09 ± 11.67	45.22 ± 11.37	41.33 ± 11.98	**0.003**	**0.028**
Cingulate_Post_L	55.1 ± 17.74	56.7 ± 17.18	51.22 ± 18.54	**0.007**	**0.028**
Cingulate_Post_R	48.42 ± 14.82	49.58 ± 14.69	45.62 ± 14.81	**0.019**	**0.044**
Precuneus_L	46.57 ± 13.79	47.78 ± 13.75	43.63 ± 13.49	**0.008**	**0.028**
Precuneus_R	46.24 ± 13.64	47.33 ± 13.49	43.62 ± 13.7	**0.017**	**0.044**
Thalamus_L	42.1 ± 10.32	42.49 ± 10.34	41.17 ± 10.25	0.264	0.284
Thalamus_R	42.99 ± 10.03	43.13 ± 9.82	42.66 ± 10.55	0.684	0.684

*Continuous variables are shown as mean ± standard deviation (SD) and examined by the t-test. a, Categorical variables are shown as number (N) and percent and examined by chi-square test. FDR_BH indicates the Benjamini–Hochberg method corrected P-value. Bold indicates that the results were significant. FDR, false discovery rate; APOE, apolipoprotein E gene; MMSE, Mini-Mental State Examination; MoCA, Montreal Cognitive Assessment; SBP, systolic blood pressure; BMI, body mass index; Temporal_Mid, middletemporalgyrus; Cingulate_Post, posterior cingulate; L, left; R, right.*

### Associations of Apolipoprotein E Genotype With Cerebral Blood Flow and Cognition

**FIGURE 1:**
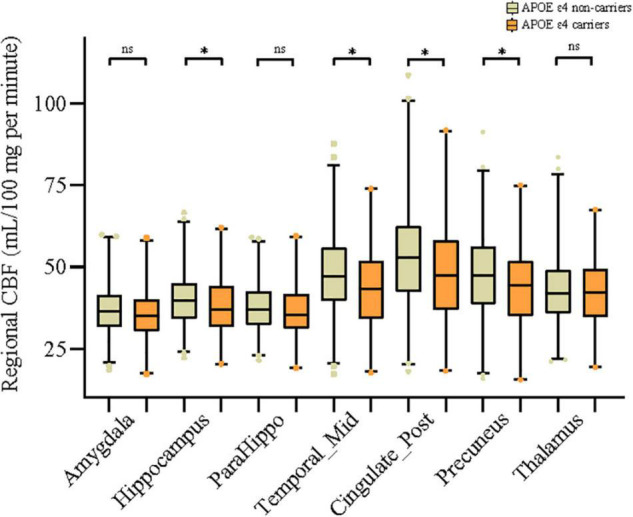
Associations of APOE ε4 allele with mean cerebral blood flow (CBF) of 7 different brain regions. Comparisons between groups were analyzed using *t*-tests. The asterisks mean significant, and ns indicates non-significant.

As can be seen in Figure 1, in the general study population, mean CBF of middle temporal gyrus (*P* = 0.004), hippocampus (*P* = 0.036), posterior cingulate (*P* = 0.009), and precuneus (*P* = 0.011) was significantly higher in the APOE ε4 non-carriers than in carriers. Similarly, as presented in Table 1, APOE ε4 carriers had lower value of all the above regional CBF in both left-hemisphere and right-hemisphere than non-carriers (all *P* < 0.05). APOE genotype was associated with CBF of amygdala (*P* = 0.04), hippocampus (*P* = 0.03), middle temporal gyrus (*P* = 0.02), posterior cingulate (*P* = 0.02), and precuneus (*P* = 0.04) but not in parahippocampal gyrus (*P* = 0.126) and thalamus (*P* = 0.593). Besides, comparison of the CBF among different APOE allele types is shown in Supplementary Table 1.

As shown in Table 2, APOE genotype correlated negatively with MoCA (β = −0.215, *P* < 0.001), after adjusting for age, gender, and years of education (model 1). Additionally, adjusting for SBP and BMI (model 2), the association of APOE and MoCA became weaker (β = −0.167, *P* = 0.002), indicating that blood pressure and BMI are important risk factors for cognition. Besides, the APOE genotype was significantly associated with lower mean CBFs of the amygdala, hippocampus, parahippocampal gyrus, middle temporal gyrus, posterior cingulate, precuneus, and thalamus (all *P* < 0.001). In model 3, as for model 2 and additionally adjusting for APOE genotype, CBF of different brain areas was positively correlated with MoCA (β range from 0.193 to 0.454, all *P* < 0.001). Among these, the most significantly associated region was middle temporal gyrus (β = 0.454, *P* < 0.001), followed by posterior cingulate (β = 0.447, *P* < 0.001) and precuneus (β = 0.438, *P* < 0.001). Subgroup analysis was performed according to age, all but the relationship of thalamic CBF with cognition in the late-life group remained, as in Supplementary Table 2. In addition, correlation values were higher in the midlife group than that in the late-life group.

**TABLE 2:** Associations of cognition with APOE and different regional CBF.

	Model 1		Model 2		Model 3		Model 4	
	β	*P*-value	β	*P*-value	β	*P*-value	β	*P*-value
APOE genotype	–4.542	< 0.001	–0.215	< 0.001	–0.167	0.002	–0.167	0.002
Amygdala	0.250	< 0.001	0.179	< 0.001	0.200	< 0.001	0.193	< 0.001
Hippocampus	0.278	< 0.001	0.245	< 0.001	0.293	< 0.001	0.286	< 0.001
Parahippocampal gyrus	0.294	< 0.001	0.219	< 0.001	0.268	< 0.001	0.264	< 0.001
Middle temporal gyrus	0.311	< 0.001	0.448	< 0.001	0.458	< 0.001	0.454	< 0.001
Posterior cingulate	0.219	< 0.001	0.403	< 0.001	0.454	< 0.001	0.447	< 0.001
Precuneus	0.260	< 0.001	0.420	< 0.001	0.443	< 0.001	0.438	< 0.001
Thalamus	0.146	< 0.001	0.182	< 0.001	0.214	< 0.001	0.214	< 0.001

*Model 1: univariate linear regression of the relationship between cognition and variables in the first column.*

*Model 2: adjusting for age, sex, and years of education.*

*Model 3: adjusting for age, sex, years of education, systolic blood pressure, and BMI.*

*Model 4: adjusting for age, sex, years of education, systolic blood pressure, BMI, and APOE.3*

*APOE, apolipoprotein E gene; CBF, cerebral blood flow; BMI, body mass index.*

To further explore the influence of the dominant hand hemisphere, we performed a subgroup analysis of the left and right hemispheres. When the left CBF and the right CBF were simultaneously included in the linear regression model, as shown in Table 3, the significances of correlations between MoCA and right amygdala, right hippocampus, right parahippocampal gyrus, right middle temporal gyrus, and right precuneus were lost (DELETED).

**TABLE 3:** Associations of CBF with cognition (DELETED).

	R^2^	*t*	*B*	95% CI		*P*-value
				Lower	Upper	
Amygdala_L	0.323	2.285	0.228	0.032	0.425	**0.023**
Amygdala_R		–0.520	–0.055	–0.262	0.152	0.603
Hippocampus_L	0.347	2.443	0.318	0.062	0.574	**0.015**
Hippocampus_R		–0.527	–0.064	–0.304	0.176	0.599
Parahippocampal gyrus _L	0.338	2.801	0.387	0.115	0.659	**0.005**
Parahippocampal gyrus _R		–1.096	–0.135	–0.377	0.108	0.274
Middle temporal gyrus _L	0.450	4.127	0.260	0.136	0.384	** < 0.001**
Middle temporal gyrus _R		0.143	0.011	–0.135	0.157	0.886
Posterior cingulate _L	0.461	6.075	0.307	0.207	0.406	** < 0.001**
Posterior cingulate _R		–2.310	–0.140	–0.260	–0.021	**0.022**
Precuneus_L	0.439	4.007	0.313	0.159	0.467	** < 0.001**
Precuneus_R		–0.882	–0.070	–0.226	0.086	0.379
Thalamus_L	0.341	3.812	0.355	0.172	0.538	** < 0.001**
Thalamus_R		–2.264	–0.208	–0.389	–0.027	**0.024**

*All models were adjusted for age, gender, education, APOE genotype, systolic blood pressure, BMI, and CBF of the specific brain area (left and right). Bold indicates that the results were significant (P < 0.05). L, left; R, right; CBF, cerebral blood flow.*

### Mediation Analysis Identified Indirect Effects Through Cerebral Blood Flow in the Associations of Apolipoprotein E Genotype With Cognition

**FIGURE 2:**
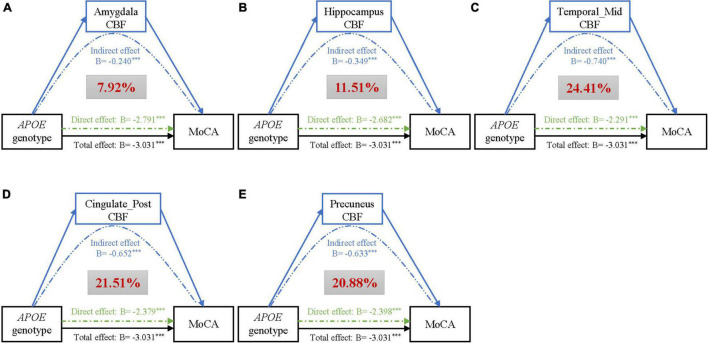
Mediation analyses of CBF of different brain regions **(A–E)** on the association of APOE genotype with MoCA, adjusting for age, gender, and education. Black lines show the total effect of APOE genotype on MoCA, green lines show the direct effect (i.e., without mediation), and blue lines depict the CBF mediation effect. Path weights are presented in *B*-values (unstandardized coefficients), and the asterisks mean that the indirect/direct/total effect is significant. Significance was determined using bootstrapping with 5,000 iterations. Additionally, the red figure within the gray box represents the proportion of mediation. Abbreviations: CBF, cerebral blood flow; MoCA, Montreal Cognitive Assessment; ParaHippo, parahippocampal gyrus; Temporal_Mid, middletemporalgyrus; Cingulate_Post, posterior cingulate.

The above findings demonstrated that there may be possible pathobiological pathways leading from APOE genotype to impaired cognition. We identified CBF of 5 regions mediating the effect of APOE genotype on cognition, after correcting for age, gender, education (refer to Figure 2). Of them, middle temporal gyrus (β_*IE*_ = −0.74, 95% CI_*IE*_ = −0.56 to −0.93), posterior cingulate (β_*IE*_ = −0.65, 95% CI_*IE*_ = −0.48 to −0.84), and precuneus (β_*IE*_ = −0.63, 95% CI_*IE*_ = −0.47 to −0.79) showed strong mediating effects, accounting for 24.41%, 21.51%, and 20.88% of the total effects of APOE genotype on cognition, respectively. Besides, CBF of the hippocampus (Proportion _*IE*_ = 11.51%) and amygdala (Proportion _*IE*_ = 7.92%) was also a potential modulator of APOE. Results of the left and right hemispheres are congruent with the results in Figure 2, and we found that brain regions of the left side have a stronger mediation than the right side (refer to Supplementary Figure 1).

## Discussion

In this prospective cohort study, three main findings were summarized as follows: (1) APOE ε4 carriers had lower perfusion in multiple brain areas compared with non-carriers; (2) cerebral perfusion had a positive association with cognition, particularly for the left (dominant) hemisphere; (3) APOE was related to cognition through a CBF-mediated pathway. Taken together, our results clearly demonstrated that APOE genotypes could associate not only with cognition but also with cerebral perfusion. Cerebral perfusion of multiple brain regions could mediate the influences of APOE on cognition, suggesting the potentially causal connections between APOE and neurodegenerative changes in the brain.

Consistent with previous theoretical work, we found that APOE ε4 carriers had reduced CBF, (Hays et al., 2016, 2019) especially in the middle temporal gyrus, hippocampus, posterior cingulate, and precuneus. In addition, the BLSA study reported that the carriers had a more rapid cerebral perfusion decline than that of non-carriers (Beason-Held et al., 2007). One longitudinal study of cognitively unimpaired older individuals showed that the hippocampus, posterior cingulate, and precuneus declined faster than other gray matter regions (McKiernan et al., 2020; Wang et al., 2021), and the heterogeneity in different brain regions may result from different causative mechanisms. A cross-sectional study from the PREVEBT-Dementia cohort also concluded that the compensatory hyperperfusion would occur at the early stages of neurodegeneration, conversely decreasing CBF at the subclinical phase of AD (McKiernan et al., 2020). During the spectrum of AD, relationships between APOE ε4 and CBF were incongruent. CBF was decreased in CN APOE ε4 carriers (Kim et al., 2013; Michels et al., 2016), but others were not (Wierenga et al., 2012; McKiernan et al., 2020). In addition, for MCI APOE ε4 carriers, findings were also contradictory (Wierenga et al., 2012; Kim et al., 2013).

However, the precise mechanisms underlying the effects of APOE on CBF and cognition are poorly defined. A study in ApoE-4 targeted replacement mice demonstrated that reduced resting CBF of APOE ε4 carriers was associated with vascular rarefaction rather than the slow velocity of a microvascular red blood cell (Koizumi et al., 2018). Besides, Østergaard et al. (2013) summarized that APOE ε4 can increase the likelihood of heterogeneity of capillary blood flow, reducing CBF, ultimately resulting in oxidative stress, activation of inflammatory pathways, and neurodegeneration. A randomized, double-blinded, placebo-controlled crossover study found that vascular function was impaired in ε4 carriers, and the peak time and magnitude of the blood oxygenation level-dependent hemodynamic response to breath-hold significantly decreased with age (Rasmussen et al., 2019). In addition, functional magnetic resonance imaging studies show that APOE ε4 carriers experienced reduced cerebrovascular reactivity, indicating highly sensitive to hypoperfusion and hypoxia (Suri et al., 2015; Koizumi et al., 2018). This conclusion is supported by preclinical and human studies, suggesting that the APOE ε4 allele was associated with higher oxidative stress and a higher pro-inflammatory state (Jofre-Monseny et al., 2008). A multimodal meta-analysis noted that APOE ε4 carriers presented a higher risk of developing white matter hyperintensity (Schilling et al., 2013), which had been widely accepted that was clearly associated with cognition decline (Wang et al., 2020).

The mediating finding in our study provided first that the causal relationship between APOE and cognition can be explained in terms of CBF. Mouse models carrying the APOE ε4 also experienced reduced CBF, and glucose metabolism and rapamycin could rescue cerebrovascular functions, CBF and incipient learning, and memory deficits in young ApoE4 mice (Lin et al., 2017). The above results increased our belief that the CBF was a mediator. Further experimental validation of this causality was required *in vitro* and *in vivo*. However, whether APOE ε4 is beneficial or aggravating remains controversial and seems to depend on the age. For example, Mondadori et al. (2007) and McKiernan et al. (2020) found that relative hyperperfusion and better cognition were observed in young APOE ε4 carriers. But ε4 carriers exhibited lower resting CBF in old age and increased the risk of AD (Thambisetty et al., 2010; Wierenga et al., 2013). When additional correction for age was applied to the association of CBF and cognition in our study, the effect size was much reduced especially in the elderly, indicating significantly affected by age. In addition, a few small studies (sample size < 100) revealed no significant correlation of APOE ε4 with CBF and cognition (Su et al., 2015; Matura et al., 2016).

This study had several strengths. We had a population-based design with a large sample size and imaging data. The data reported in this study will serve as a baseline for follow-up studies and be conducted to pursue an in-depth understanding in this regard. CBF was obtained by non-invasive ASL MRI, which had good agreements with quantitative CBF values derived from ^15^O-H_2_O PET (Kamano et al., 2013). Additionally, an imaging examination was performed after overnight fasting for 8 h, minimizing the effects of metabolic factors. However, several potential limitations should be addressed. First, our study was hospital-based, and the generalizability of conclusions warrants further validation in large community-based longitudinal studies. Second, ASL measurements were not corrected for partial volume effect (PVE), until now, the correction approaches are inconsistent, and the consistent benefit of adjusting for PVE has not been shown (Chappell et al., 2021). In addition, we did not consider the influence of the whole brain CBF. Currently, examination of serum or cerebrospinal fluid biomarkers related to neurodegenerative diseases has been not performed in the CIBL cohort. Besides, multi-delay ASL was required to verify these results, due to the effect of different arterial transit times.

## Conclusion

Results demonstrated cerebral perfusion as an independent risk factor not only for cognitive impairments but also an important mediator for the effects of APOE genotype on the cognitive deficiency. The adverse genetic influence of APOE ε4 on cognition may be moderated by improved cerebral vasculature and CBF.

## Data Availability Statement

The raw data supporting the conclusions of this article will be made available by the authors, without undue reservation.

## Ethics Statement

The studies involving human participants were reviewed and approved by the Research Ethics Committee of Beijing Tiantan Hospital. The patients/participants provided their written informed consent to participate in this study.

## Author Contributions

JX, XL, and Y-LW conceived and designed the study. Y-LW contributed to generation of the manuscript. All authors contributed to the editing of the manuscript.

## Conflict of Interest

The authors declare that the research was conducted in the absence of any commercial or financial relationships that could be construed as a potential conflict of interest.

## Publisher’s Note

All claims expressed in this article are solely those of the authors and do not necessarily represent those of their affiliated organizations, or those of the publisher, the editors and the reviewers. Any product that may be evaluated in this article, or claim that may be made by its manufacturer, is not guaranteed or endorsed by the publisher.
