# Supplementary material for: Mediation of the APOE Associations With Cognition Through Cerebral Blood Flow: The CIBL Study
Source: Front Aging Neurosci. 2022 Jun 30;14:928925. doi: 10.3389/fnagi.2022.928925 (PMC9279129; doi:10.3389/fnagi.2022.928925)
Supplement: Supplementary file 1 [file Image_1.pdf]

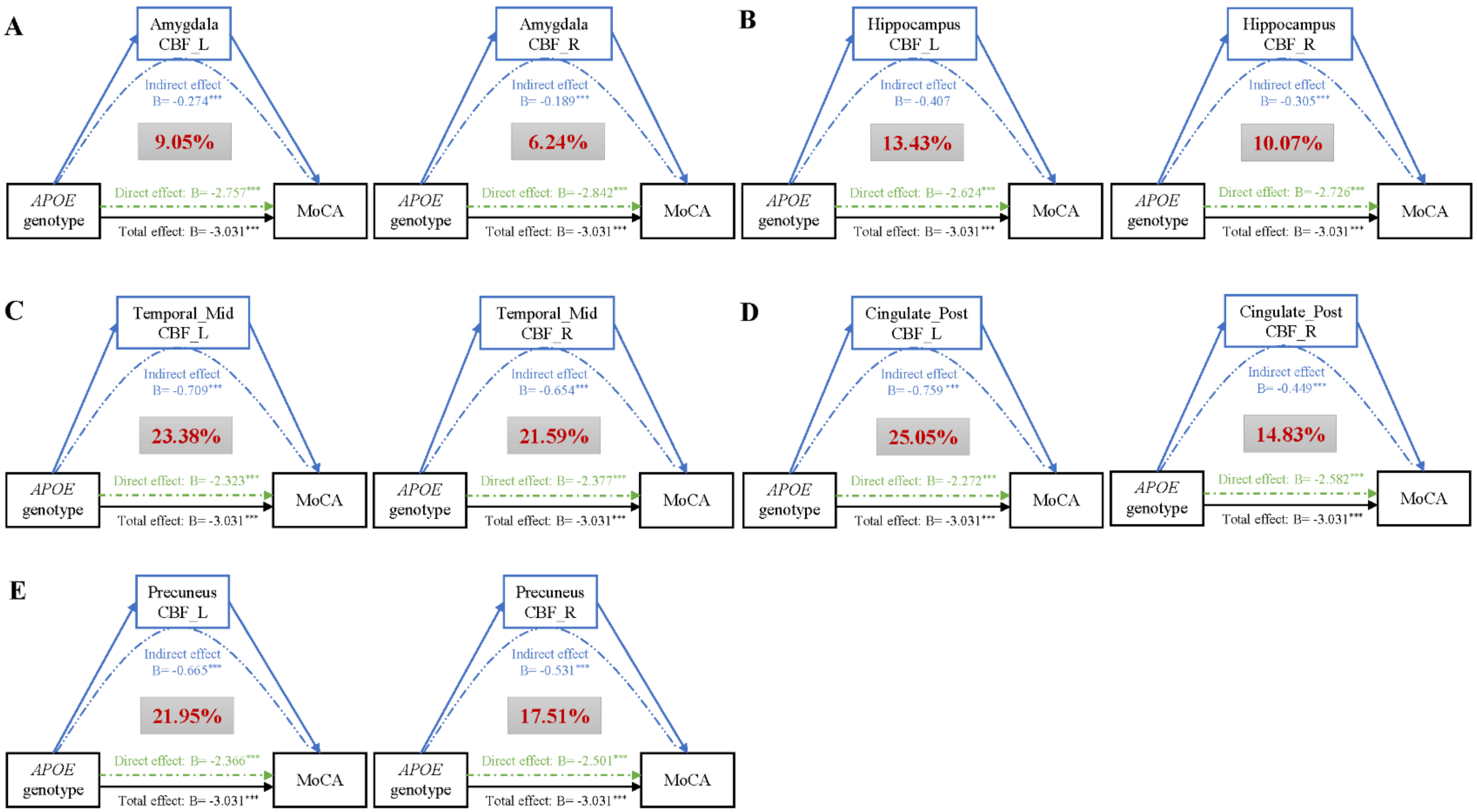

eFigure 1. Mediation analyses of CBF of 5 significant brain regions (left/right) (A-E) on the association of APOE genotype with MoCA, adjusting for age, gender and education.
