# Supplementary material for: Mediation of the APOE Associations With Cognition Through Cerebral Blood Flow: The CIBL Study
Source: Front Aging Neurosci. 2022 Jun 30;14:928925. doi: 10.3389/fnagi.2022.928925 (PMC9279129; doi:10.3389/fnagi.2022.928925)
Supplement: Supplementary file 2 [file Table_1.docx]

eTable1. Comparison of the CBF among different APOE allele type.

|  | All subjects |  | APOE ε4 -/- |  | APOE ε4 -/+ |  | APOE ε4 +/+ | P value |
| --- | --- | --- | --- | --- | --- | --- | --- | --- |
|  | (N=369) |  | (N=261) |  | (N=92) |  | (N=16) |  |
| Amygdala_L | 37.12±7.89 |  | 37.61±7.7 |  | 36.22±8.51 |  | 34.39±6.76 | 0.129 |
| Amygdala_R | 35.76±7.74 |  | 36.27±7.74 |  | 34.56±7.97 |  | 34.28±5.5 | 0.139 |
| Hippocampus_L | 39.92±8.34 |  | 40.5±8.07 |  | 38.87±9.17 |  | 36.7±6.48 | 0.078 |
| Hippocampus_R | 39.27±8.87 |  | 39.88±8.46 |  | 37.87±10.1 |  | 37.35±7.13 | 0.119 |
| ParaHippocampal_L | 36.54±7.37 |  | 36.86±7.24 |  | 35.81±7.8 |  | 35.51±7.05 | 0.425 |
| ParaHippocampal_R | 38.2±8.25 |  | 38.69±8.04 |  | 37.05±9.09 |  | 36.85±5.99 | 0.207 |
| Temporal_Mid_L | 48.8±13.26 |  | 49.99±12.96 |  | 45.38±13.92 |  | 48.98±11.53 | **0.016** |
| Temporal_Mid_R | 44.09±11.67 |  | 45.22±11.37 |  | 40.62±12.06 |  | 45.44±10.94 | **0.004** |
| Cingulate_Post_L | 55.1±17.74 |  | 56.7±17.18 |  | 50.98±18.99 |  | 52.65±16.18 | **0.024** |
| Cingulate_Post_R | 48.42±14.82 |  | 49.58±14.69 |  | 45.27±15.31 |  | 47.63±11.77 | 0.055 |
| Precuneus_L | 46.57±13.79 |  | 47.78±13.75 |  | 43.07±13.92 |  | 46.88±10.44 | **0.018** |
| Precuneus_R | 46.24±13.64 |  | 47.33±13.49 |  | 43.09±14.19 |  | 46.63±10.25 | **0.037** |
| Thalamus_L | 42.1±10.32 |  | 42.49±10.34 |  | 41.06±10.57 |  | 41.76±8.44 | 0.521 |
| Thalamus_R | 42.99±10.03 |  | 43.13±9.82 |  | 42.3±11.04 |  | 44.77±7.07 | 0.608 |

Continuous variables are shown as mean ± standard deviation (SD) and examined by ANOVA.
