# Supplementary material for: Mediation of the APOE Associations With Cognition Through Cerebral Blood Flow: The CIBL Study
Source: Front Aging Neurosci. 2022 Jun 30;14:928925. doi: 10.3389/fnagi.2022.928925 (PMC9279129; doi:10.3389/fnagi.2022.928925)
Supplement: Supplementary file 3 [file Table_2.docx]

eTable 2. Associations of CBF with cognition in mid-life and late-life

|  | Mid-life | | | Late-life | | |
| --- | --- | --- | --- | --- | --- | --- |
|  | B | t | P value | B | t | P value |
| Amygdala | 0.266 | 2.685 | 8.38E-03 | 0.170 | 2.432 | 1.63E-02 |
| Hippocampus | 0.345 | 3.938 | 1.44E-04 | 0.205 | 2.990 | 3.34E-03 |
| Parahippocampal gyrus | 0.358 | 3.787 | 2.49E-04 | 0.194 | 2.496 | 1.38E-02 |
| Middle temporal gyrus | 0.410 | 8.044 | 1.09E-12 | 0.192 | 4.196 | 4.97E-05 |
| Posterior cingulate | 0.306 | 7.864 | 2.76E-12 | 0.142 | 4.002 | 1.05E-04 |
| Precuneus | 0.332 | 7.297 | 4.85E-11 | 0.166 | 3.928 | 1.38E-04 |
| Thalamus | 0.301 | 3.560 | 5.49E-04 | 0.095 | 1.712 | **8.93E-02** |

Bold indicated that the results were not significant (p> 0.05)
